# Supplementary material for: Impaired cerebellar Purkinje cell potentiation generates unstable spatial map orientation and inaccurate navigation
Source: Nat Commun. 2019 May 21;10:2251. doi: 10.1038/s41467-019-09958-5 (PMC6529420; doi:10.1038/s41467-019-09958-5)
Supplement: Supplementary file 1 — Supplementary Information [file 41467_2019_9958_MOESM1_ESM.pdf]

Supplementary Information

Impaired cerebellar Purkinje cell potentiation generates unstable spatial map orientation and inaccurate navigation

Lefort *et al.*

A

Task	Measure	Controls (N=36)	L7-PP2B (N=24)	*t-test (p)*
**Anxiety**	% Time in open arms	45.9 ± 4.5	45.3 ± 5.4	0.94
**Spontaneous locomotor activity**	Speed (cm.s^{-1})	13.8 ± 0.3	13.5 ± 0.4	0.56
	Travelled distance (m)	27.5 ± 1.7	26.0 ± 1.7	0.57
	Rearing number	32.9 ± 3.3	27.1 ± 3.5	0.25
**Motor coordination**	Walking time (min)	1.4 ± 0.1	1.3 ± 0.1	0.65
	Slip frequency	5.5 ± 0.5	6.2 ± 0.6	0.32
**Static balance**	Falling latency (s)	125.5 ± 6.4	129.1 ± 7.7	0.72
**Dynamic balance**	Falling latency (s)	180.0	180.0	-
	Travelled distance (m)	18.5 ± 1.0	16.9 ± 1.2	0.32
	Speed (cm.s^{-1})	11.6 ± 0.3	10.8 ± 0.5	0.16

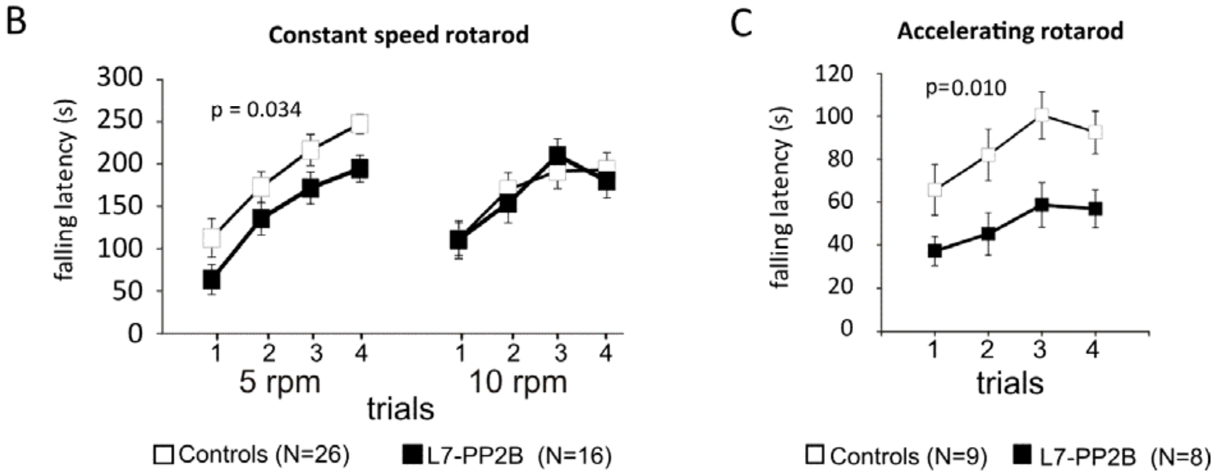

Supplementary Figure 1. Analyses of mice general sensori-motor properties and motor adaptation abilities. a Table showing the basic sensori-motor abilities of controls and L7-PP2B mice. b-c Motor adaptation performances expressed as a latency to fall from a rod rotating at a constant (b) or accelerating (c) speed in both control and L7-PP2B mice (constant speed, repeated measure ANOVA, genotype, 5rpm $F_{(1,40)}$= 4.82, p= 0.034 ; 10 rpm $F_{(1,40)}$= 0.01, p=0.92; accelerating protocol, repeated measure ANOVA, genotype, $F_{(1,15)}$= 8.79, p= 0.01). Error bars represent S.E.M. N indicates the number of independent individuals.

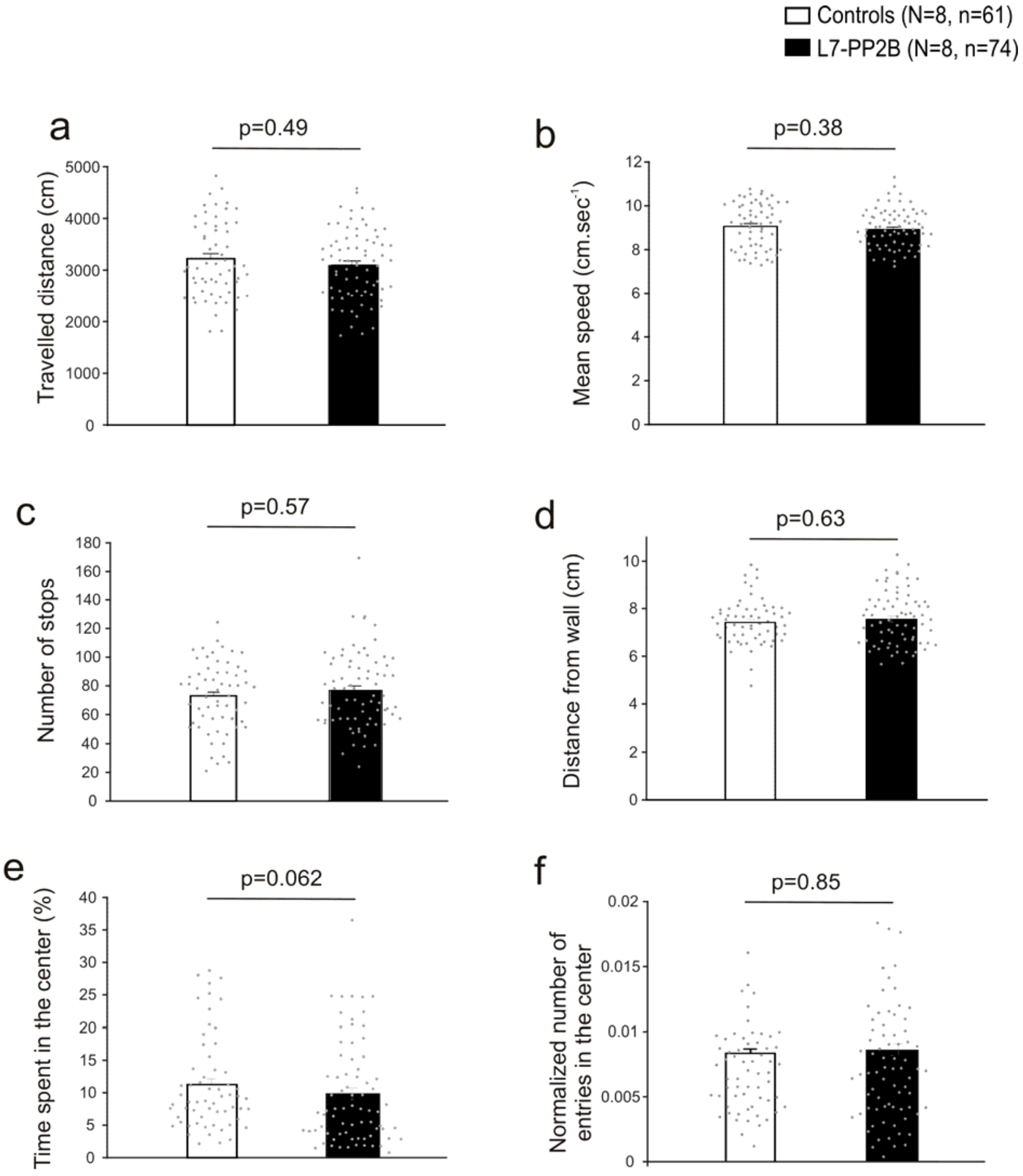

Supplementary Figure 2. Analyses of mice behavior during the first session S1 of exploration of the familiar arena. General locomotor behavior was similar between controls and L7-PP2B mice as assessed by the travelled distance (a, U=2101, p=0.49, Mann-Whitney U-test), mean speed (b, U=2057, p=0.38, Mann-Whitney U-test) and number of stops (c, U=2127, p=0.57, Mann-Whitney U-test). Exploratory behavior of L7-PP2B mice was also not different from controls as illustrated by the distance from wall (d, U=2146, p=0.63, Mann-Whitney U-test), % of time spent in the center (e, U=1834, p=0.062, Mann-Whitney U-test) or the number of entries in the center normalized by the total travelled distance (f, U=2213, p=0.85, Mann-Whitney U-test). Individual data are shown in grey, error bars represent S.E.M. N and n indicate mice and session number respectively

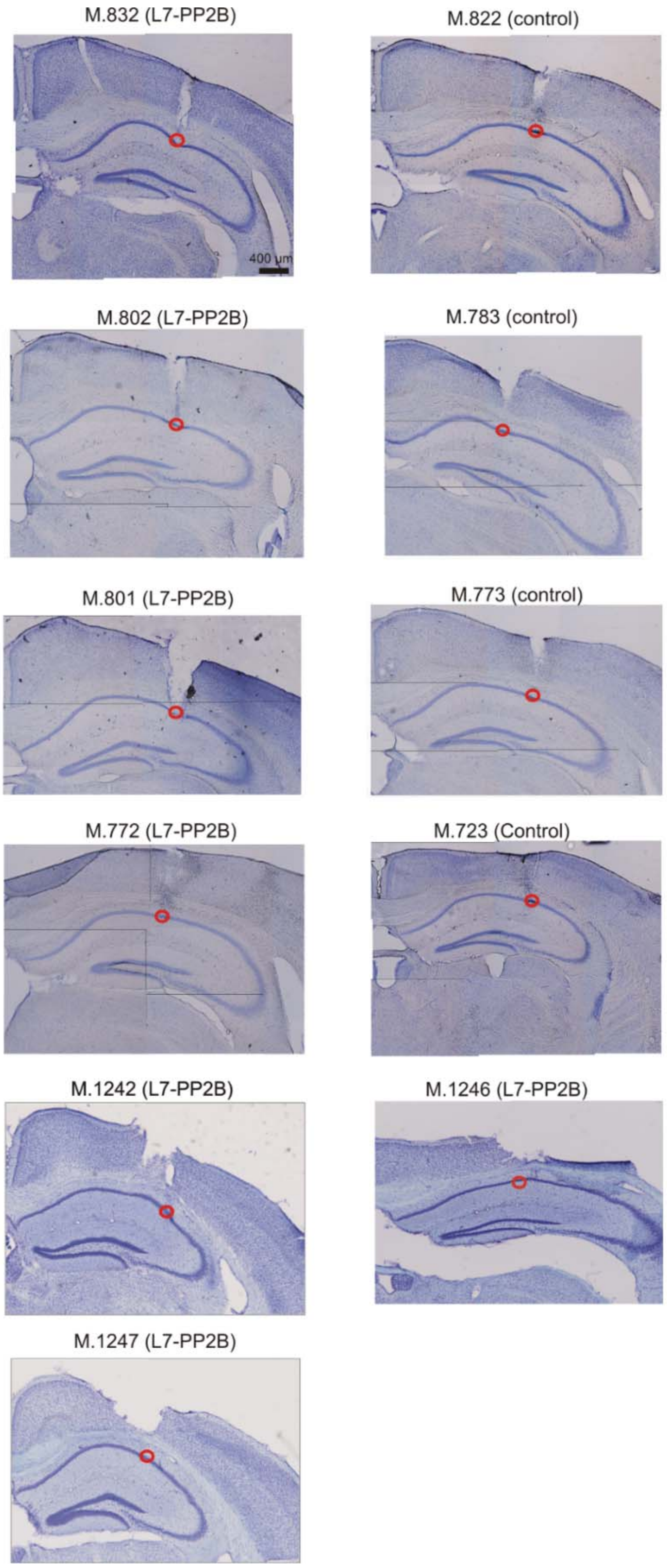

Supplementary Figure 3. Representative Nissl-stained brain sections showing recording sites (red circle) in the CA1 region of the hippocampus in 4 controls and 7 L7-PP2B mice. 4 control and 1 L7-PP2B mice were not included in the staining protocol. Scale bar: 400 µm.

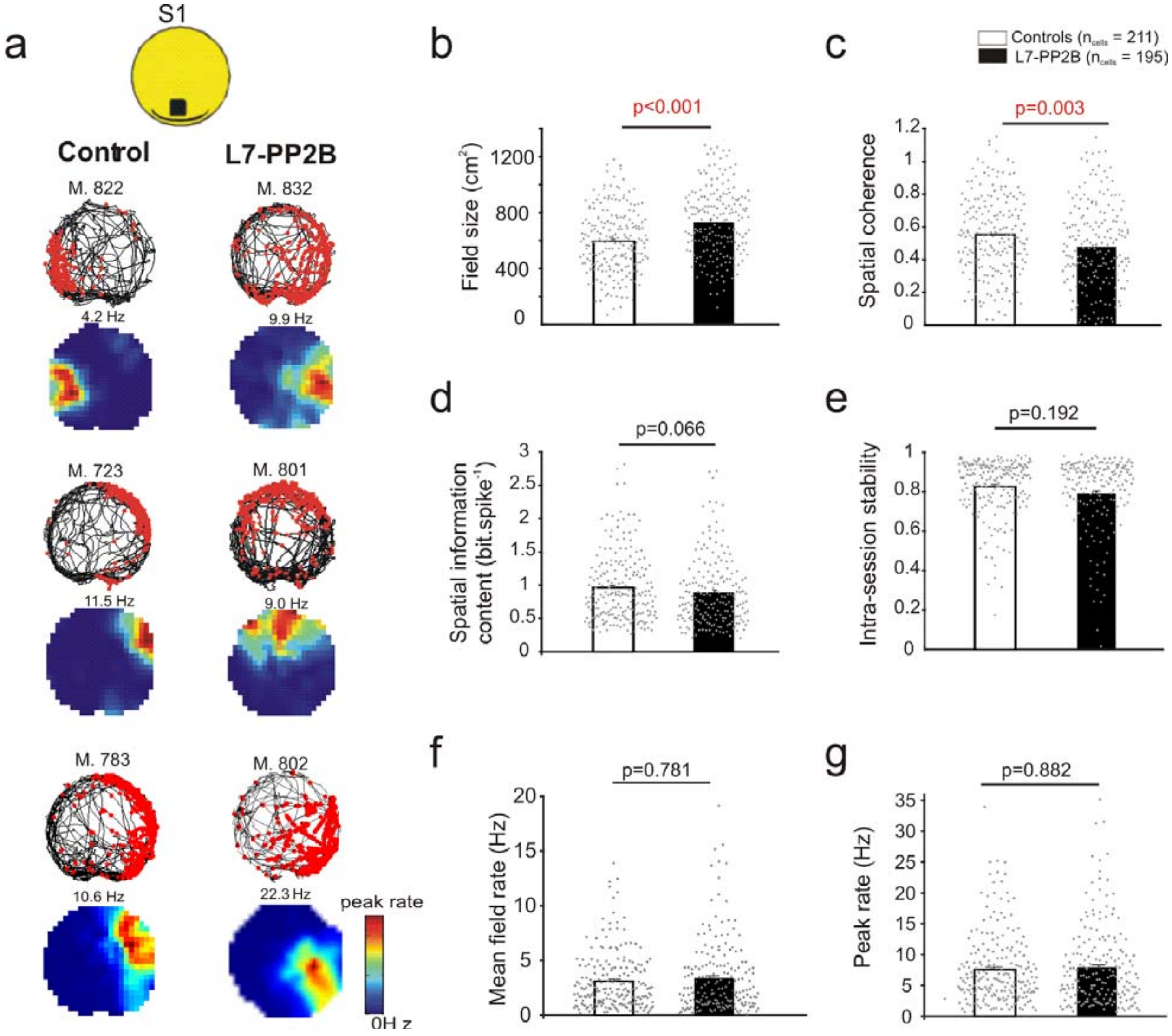

Supplementary Figure 4. Hippocampal place cells properties of L7-PP2B mice are impaired in a familiar environment. a Examples of trajectories (in black) with spike location (in red) (upper row) and color-coded rate maps (lower row) showing place cells recorded during the first session of exploration of a familiar environment. Text above trajectories indicates mouse identity. Text above the rate maps indicates the peak rate. b-g Bar plots showing the field size (b, U =14203, p<0.001, Mann-Whitney U-test), spatial coherence (c, U =17078, p=0.003, Mann-Whitney U-test), spatial information content (d, U =18402, p=0.066, Mann-Whitney U-test), intra-session stability (e, U =19031, p=0.192, Mann-Whitney U-test), mean field rate (f, U =19948, p=0.781, Mann-Whitney U-test) and peak rate (g, U =20397, p=0.882, Mann-Whitney U-test) during exploration of a familiar environment. Data from individuals cells are shown in grey, error bars represent S.E.M.

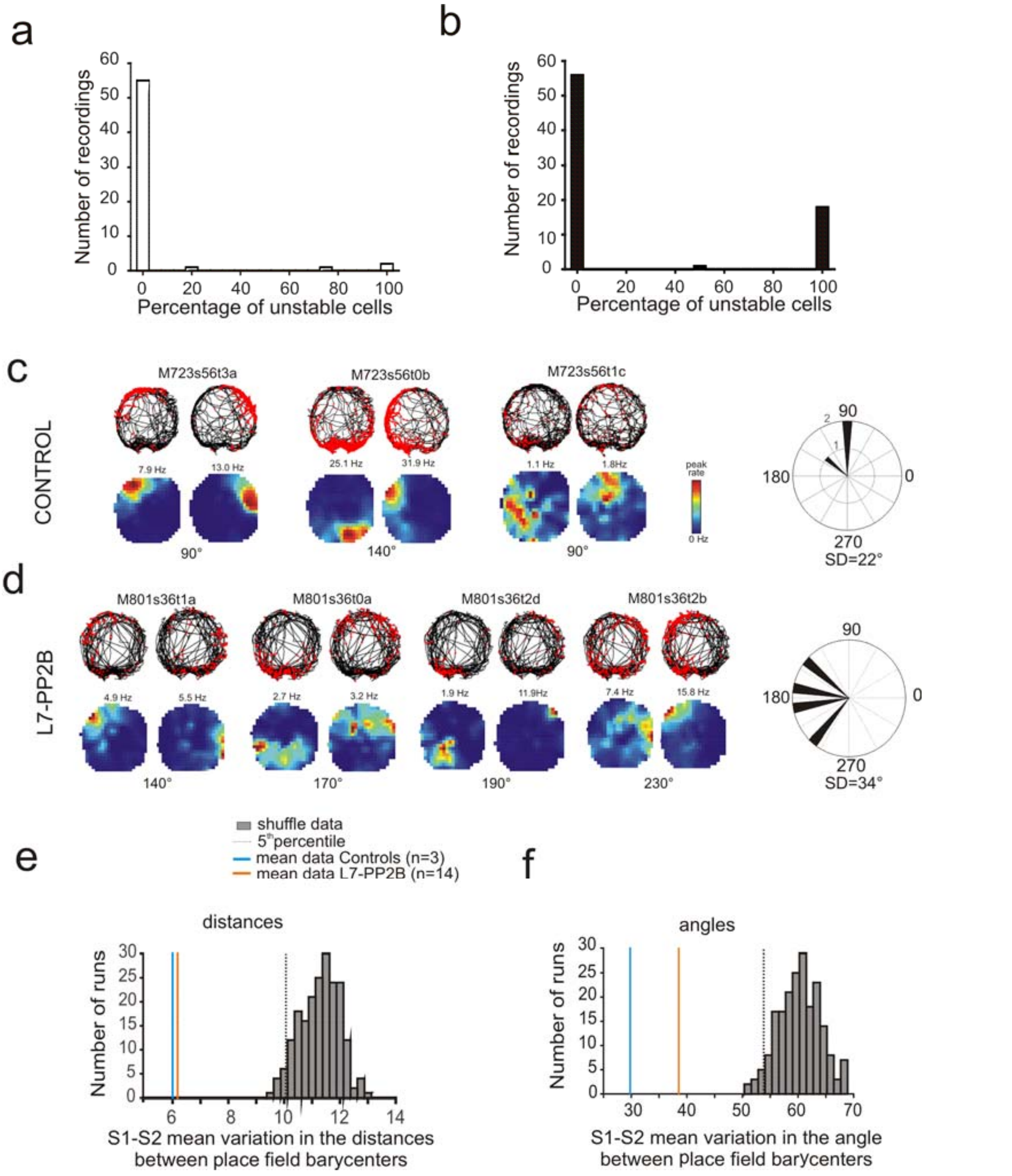

Supplementary Figure 5. Instabilities correspond to a coherent rotation of place fields a-b Histograms depicting the distribution of recording sessions in function of the percentage of unstable cells per recording for control (a) and L7-PP2B (b) mice. Recording sessions are essentially separated in two groups (0% and 100%, the other percentage being almost absent). c-d Examples of simultaneously recorded control (c) and L7-PP2B (d) place cells showing coherent place field rotation between S1 and S2. The rotation angle, computed for each place cell, is indicated between the rate maps, and reported on the polar plot on the right. Standard deviation, computed for the angles of the simultaneously recorded cells, is indicated below. The place cell ensemble from the controls contained one object cell that is not shown on the figure. Cell identity is indicated above the rate maps. The place cell ensemble from the controls contained one object cell that is not shown on the figure. Cell identity is indicated above the rate maps. e-f Analysis of the spatial relationships between place fields of simultaneously recorded cells. The mean variation of distances (e) and angles (f) of place field centroids between S1 and S2 is compared to the shuffle distribution: distributions of S1-S2 mean variation in distances (e) and angles (f) between randomly chosen place field barycenters (supplementary method). The mean variation in field barycenter distances and angles between S1 and S2 (real data) is indicated for controls (blue) and mutants (orange) indicating mean data of both groups are not random. The black dashed line highlights the 5th percentile of the shuffle distribution.

	L7-PP2B					Control
	M696	M832	M801	M802	M1247	M723
stable sessions	9	15	3	5	11	11
unstable sessions	5	1	5	3	4	3
stable cells	22	49	11	10	24	34
unstable cells	9	1	19	8	10	10
proportion unstable sessions (%)	35.7	6.3	62.5	37.5	26.7	21.4

Supplementary Figure 6. Table indicating the number of unstable sessions, unstable cells, and proportion of unstable sessions for each mouse with unstable place cells.

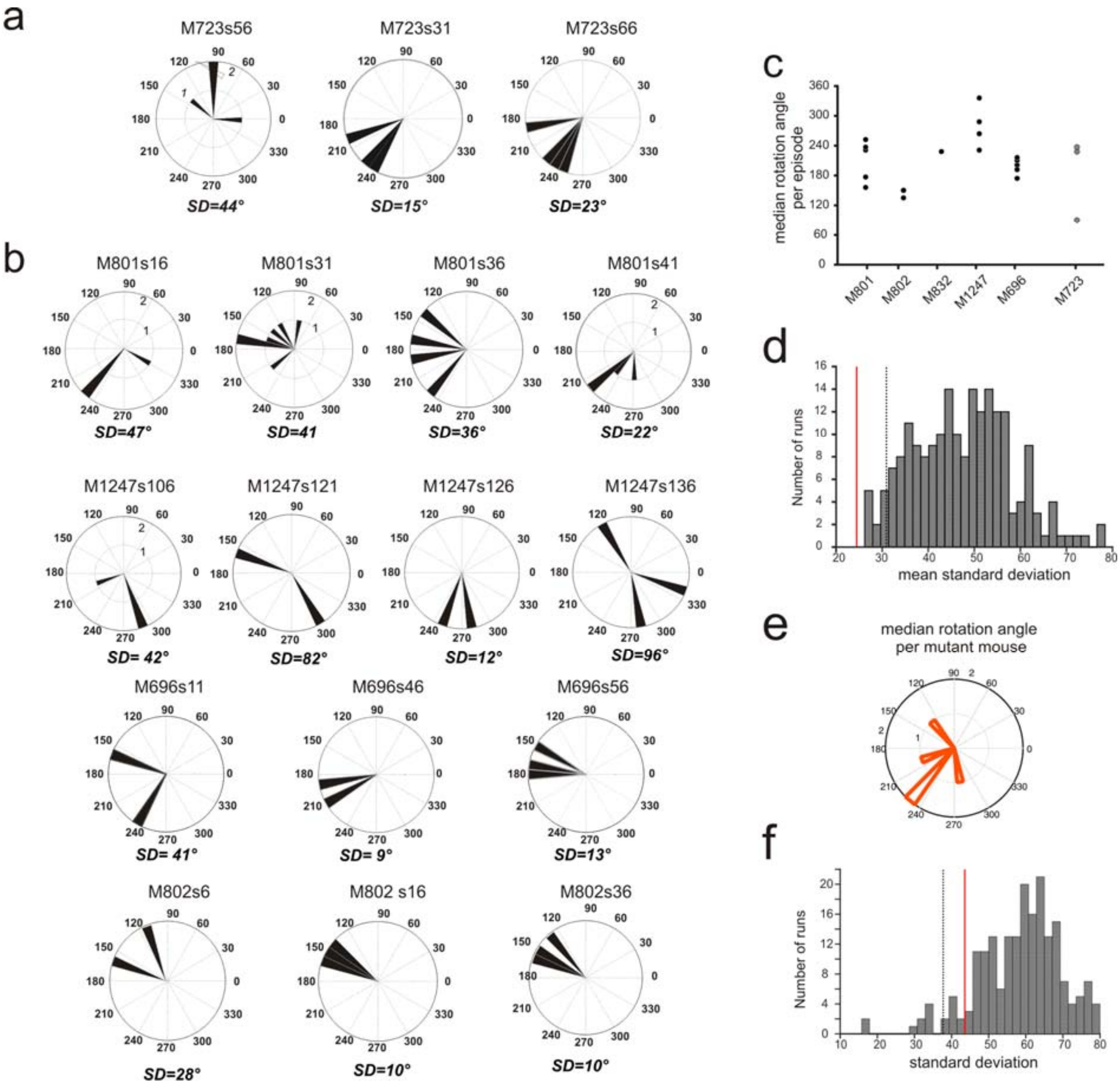

Supplementary Figure 7. a-b Polar plots illustrating unstable place cells from ensemble recordings. Each polar histogram plots the rotation angles of simultaneously recorded place cells for one instability episode occurring between familiar sessions S1 and S2 (a) shows the three instability episodes which occurred in one single control mouse and (b) the 14 instability episodes observed in L7-PP2B mice. The coherent rotation of the place fields is interpreted as map rotation. The mouse identity and session index is indicated above the plots. Standard deviation (SD) computed for each episode is indicated below the polar plots. c Distribution of the different median rotation angles observed for each episode of instability (black dots : mutant mice, grey dot : control mouse). d Quantification of the within mouse consistency for mutant mice. The mean standard deviation (SD) is compared to the distribution of mean SD computed on shuffle data. The mean SD (red line) is below the fifth percentile of the distribution (black dashed line). e Polar plot illustrating the median rotation angles per mutant mouse. f Distribution of between mouse-median directions. The standard deviation (SD) is compared to the distribution of SD computed on shuffle data. The SD (red line) is above the fifth percentile of the distribution (black dashed line). These plots illustrate that the rotation angles are globally similar between different episodes of a given individual but are variable between mice.

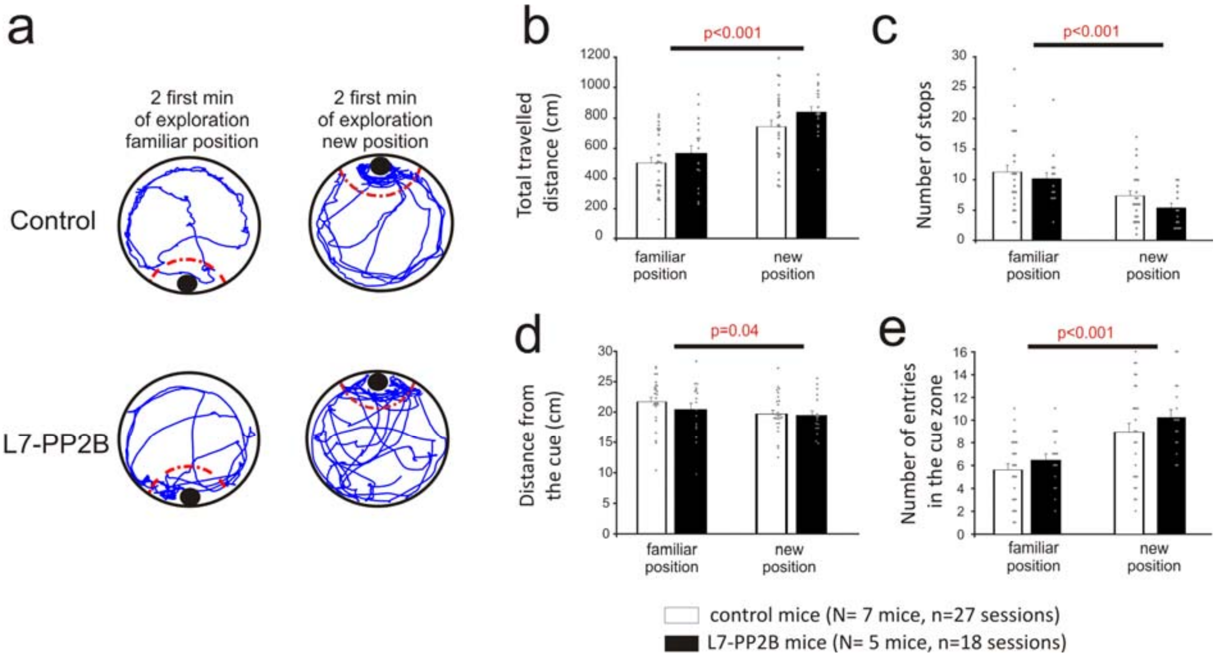

Supplementary Figure 8. Effect of a visible cue rotation on control and L7-PP2B exploratory behavior. a Examples of control and L7-PP2B trajectories before (familiar position) and after (new position) a visible displacement of the object b-e bar plots showing the total travelled distance (b), the number of stops (c) the distance from the cue (d) and the number of entries in the cue zone (e) before and after cue rotation. These data illustrate that both genotype displayed an increase in the travelled distance (b, session, $F_{(1,43)} = 46.05$, $p < 0.001$; genotype, $F_{(1,43)} = 2.67$, p=0.11; session * genotype interaction, $F_{(1,43)} = 0.57$, p=0.45), a decrease in the number of stops (c, session, $F_{(1,43)} = 16.16$, $p < 0.001$; genotype, $F_{(1,43)} = 2.46$, p=0.12; session * genotype interaction, $F_{(1,43)} = 0.17$, p=0.68), a decrease in the distance from the cue (d, session, $F_{(1,43)} = 4.5$, p = 0.040; genotype, $F_{(1,43)} = 0.803$, p=0.38; session*genotype interaction, $F_{(1,43)} = 0.29$, p=0.59) and an increase in the number of entries in the cue zone (e, session, $F_{(1,43)} = 28.9$, $p < 0.001$; genotype, $F_{(1,43)} = 2.02$, p = 0.16; session*genotype interaction, $F_{(1,43)}=0.15$, p=0.70) when the cue has moved to a new place (two-way ANOVA with repeated measures).

a

	Mean ± S.E.M. Controls (n=6)	Mean ± S.E.M. L7-PP2B (n=4)	Mann-Whitney U	Mann-Whitney p value
**locomotor activity in the dark**				
Rearing frequency (number/min)	8.5 ± 0.8	9.5 ± 0.4	7	0.35
**Dynamic balance in the dark**				
Falling latency (s)	180	180	--	--
Distance travelled (cm)	508 ± 56	494 ± 115	11	0.91
**Static balance in the dark**				
Falling latency (s)	149 ± 7	137 ± 25	10.5	0.76
**Motor coordination in the dark (rotarod)**				
**5 rpm** *walking time (s)*	128 ± 26	106 ± 18	9	0.61
**10 rpm** *walking time (s)*	19 ± 4	65 ± 44	6	0.26

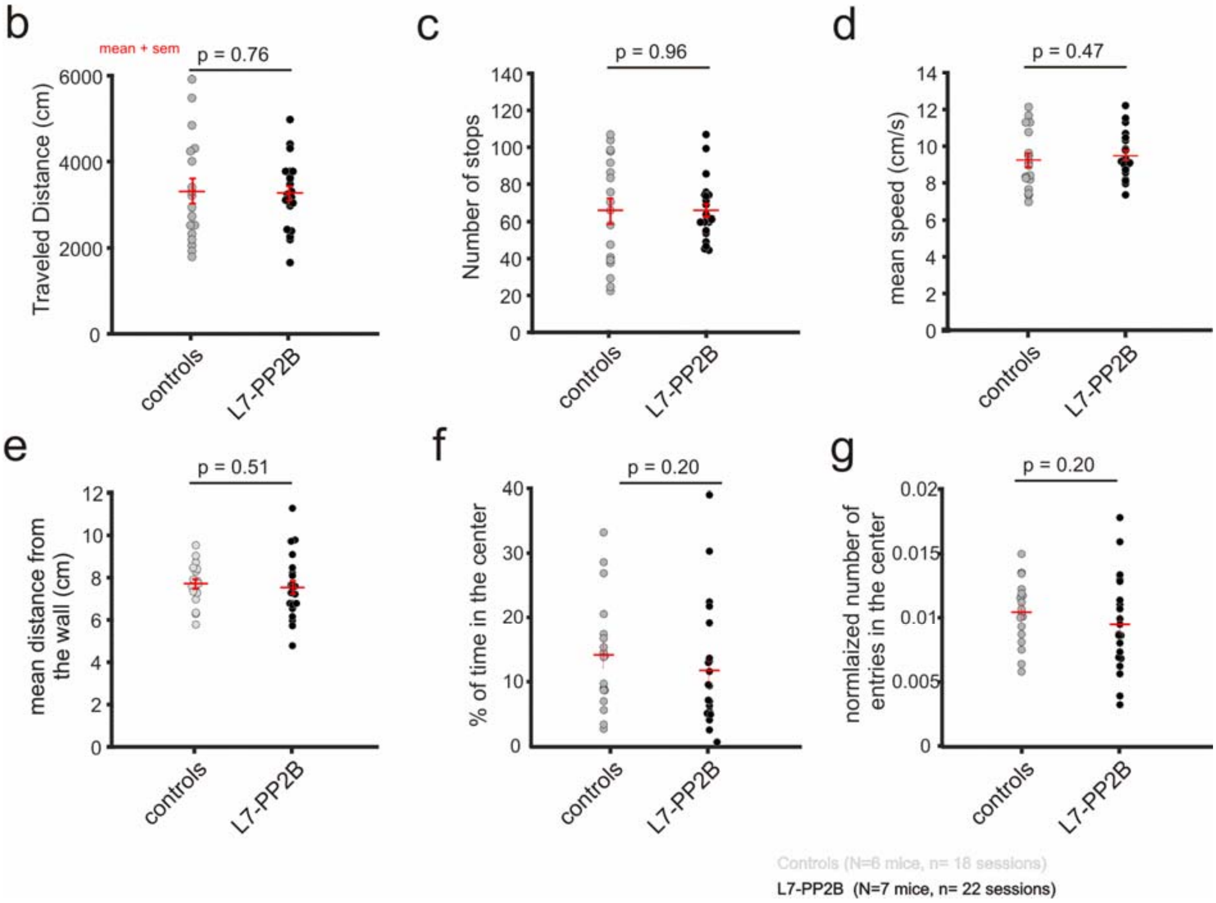

Supplementary Figure 9. Sensory-motor properties and exploratory behavior in self-motion dependent context (in the dark, no visual cue) are unaltered in L7-PP2B mice. a General sensory-motor abilities including rearing, balance and motor coordination assessed in the dark, i.e. using primarily the vestibular system, revealed no difference between control and L7-PP2B mice. b-d scatter plots showing that control and L7-PP2B mice display no difference in their general locomotor activity in the dark in the familiar arena, as illustrated by the traveled distance (b, U=186, p=0.76, Mann-Whitney U-test), the number of stops (c, U=196, p=0.96, Mann-Whitney U-test) or the mean speed (d, U=171, p=0.47, Mann-Whitney U-test). e-g The center versus periphery exploratory behavior was also similar between control and mutant mice. No difference was found in the mean distance from the wall (e, U=173, p=0.51, Mann-Whitney U-test), the percentage of time spent in the center (f, U=150, p=0.20, Mann-Whitney U-test) or the normalized number of entries in the center (g, U=150, p=0.20, Mann-Whitney U-test). Error bars represent S.E.M.

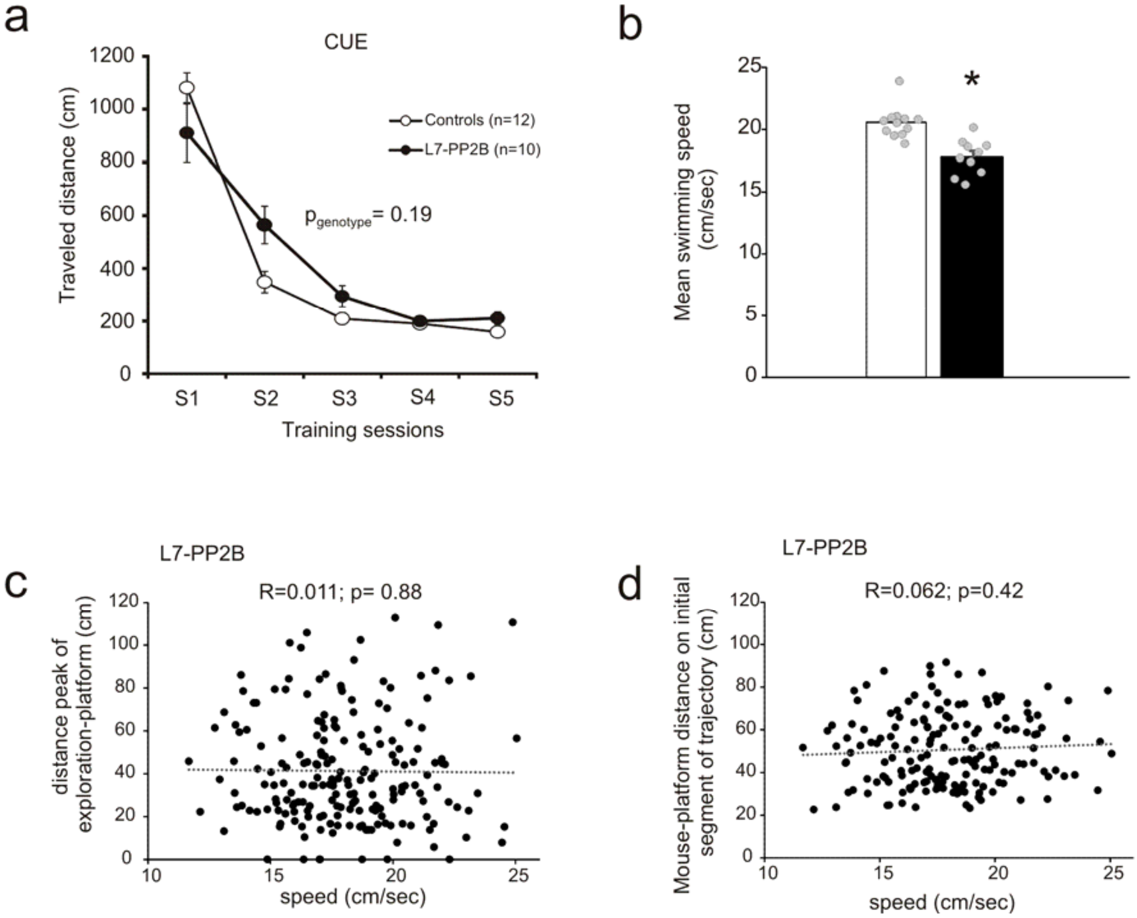

Supplementary Figure 10. Visuo-motor abilities are preserved in L7-PP2B mice. a No difference between genotypes was observed in the traveled distance to find the visible platform over the five sessions of cue training. (Repeated measure ANOVA, Cue phase $F_{(1,20)}$=1.867, $p_{genotype}$=0.19). b Mean swimming speed for L7-PP2B mice (n=10) compared to controls (n=12), (Mann-Whitney U-test, U=14, p=0.002). Individuals data are shown in grey. c-d The spatial performances of L7-PP2B are not correlated with their speed suggesting that the deficits observed in the Morris Water maze do not result from impaired motor performances. N mice = 10, n trials=200. Error bars represent S.E.M. *, p< 0.05.

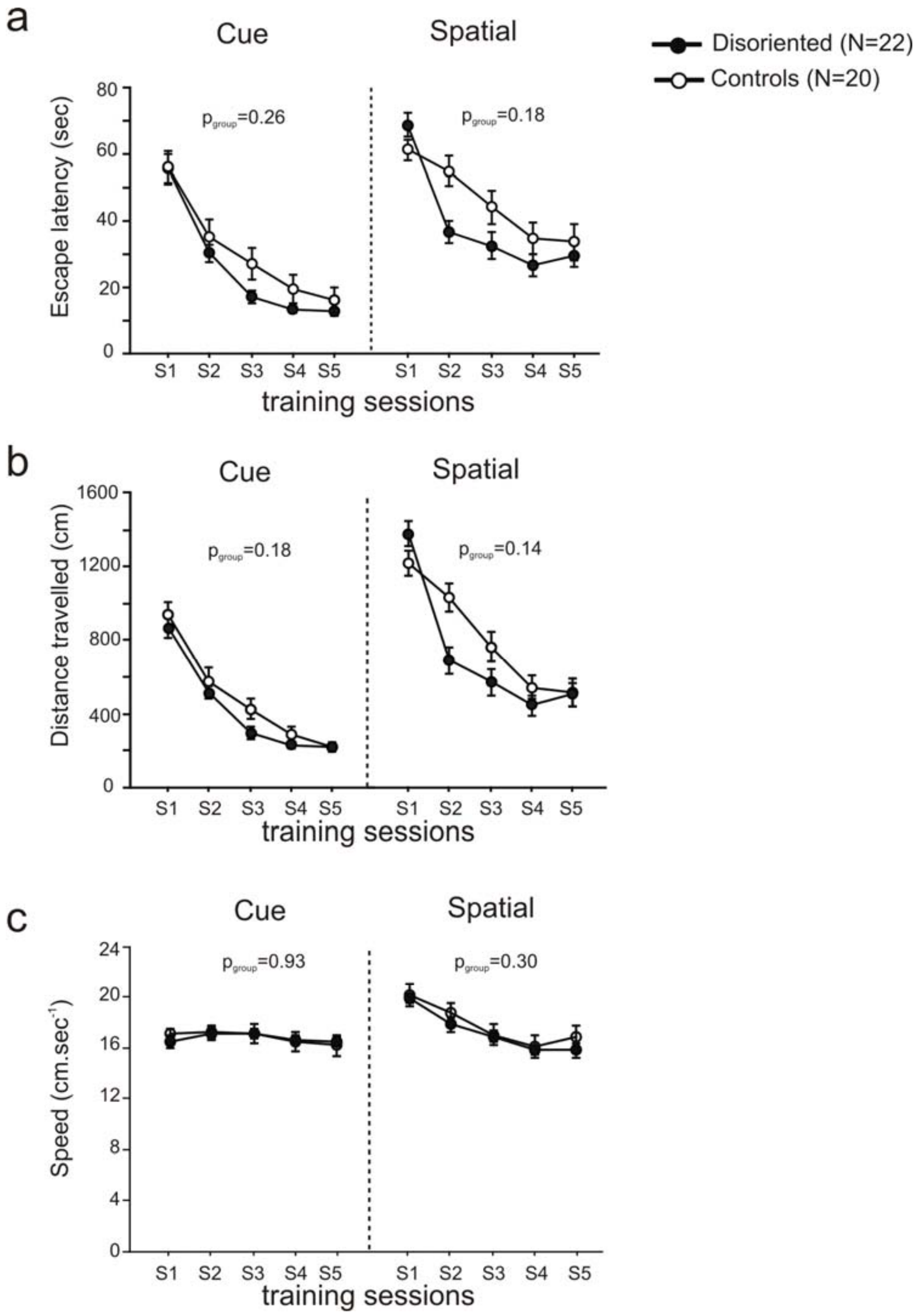

Supplementary Figure 11. Passively-disoriented mice are not impaired in the Morris Water Maze. a Escape latencies of disoriented mice are preserved in both the cue and spatial version of Morris Water Maze (repeated measure ANOVA, cue: genotype, $F_{1,40}$=1.3, p=0.26; genotype * session, $F_{4,40}$=1.2, p=0.31; spatial: genotype, $F_{1,40}$=1.88, p=0.18; genotype*sessions, $F_{4,40}$=5.36, p<0.001). b Travelled distances to the platform of disoriented mice are not impaired in both the cue and spatial version of the Morris Water Maze (repeated measure ANOVA, cue: genotype, $F_{1,40}$=1.8, p=0.18; genotype * session, $F_{4,40}$=0.7, p=0.56; spatial: genotype, $F_{1,40}$=2.3, p=0.14; genotype*sessions, $F_{4,40}$=4.59, p=0.002). c Speed of disoriented mice are similar to controls in both the cue and spatial version of the Morris Water Maze (repeated measure ANOVA, cue: genotype, $F_{1,40}$=0.009, p=0.93; genotype * session, $F_{4,40}$=0.4, p=0.81; spatial: genotype, $F_{1,40}$=1.1, p=0.30; genotype*sessions, $F_{4,40}$=0.65, p=0.63). Error bars represent S.E.M.

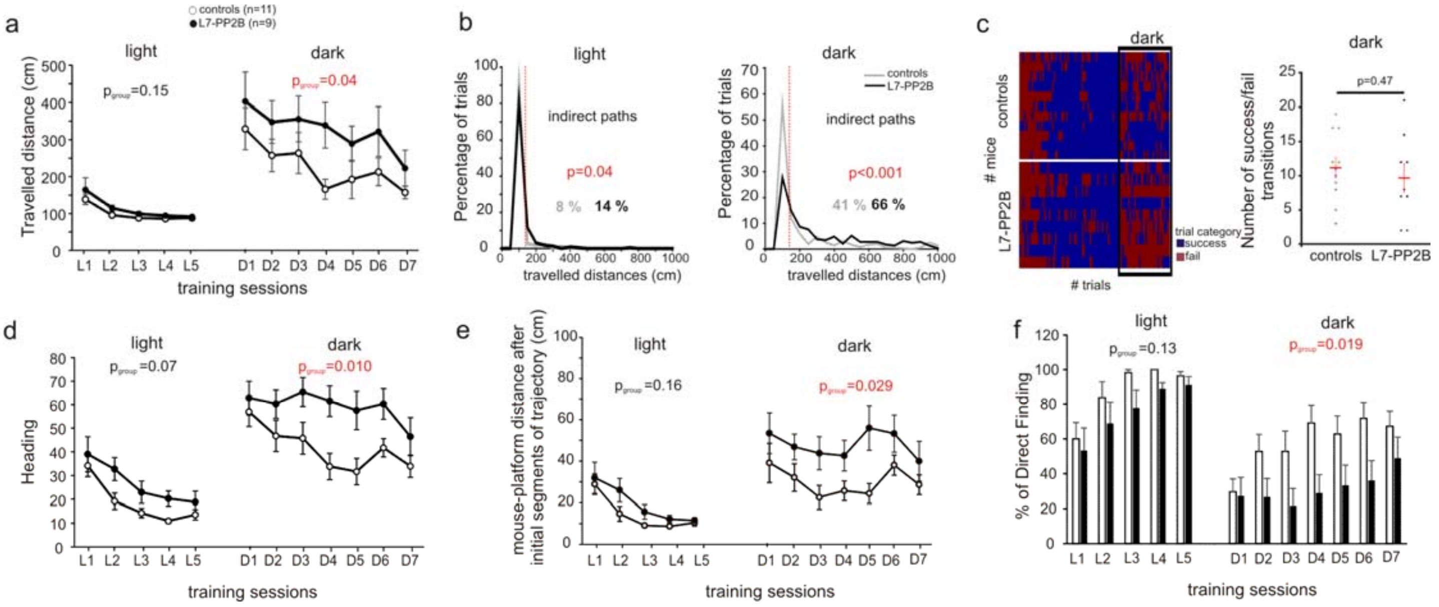

Supplementary Figure 12. Self-motion based navigation performances are impaired in L7-PP2B mice. a Travelled distances of L7-PP2B (n=9) and controls (n=11) mice showing that L7-PP2B mice performances were preserved in the light but impaired in dark conditions (repeated measure ANOVA, genotype, light: $F_{1,18}$= 2.25, p=0.15; dark: $F_{1,18}$=5.06, p= 0.037). b Distributions of travelled distances of all trials for control (grey) and L7-PP2B (black) mice. Separation of direct (short) and indirect (long) trials was made using a Gaussian fit (see methods), the threshold (138 cm) is indicated by the red dashed line. c *Left*, Color-coded representation of travelled distances for all trials indicating successful (direct, blue) and failed (indirect, brown) trials. Each line represents the performances of one mouse over the course of training. *Right*. The number of transitions between successful and failed trials is not different between L7-PP2B and control mice (p=0.47, Mann-Whitney U-test) in the dark. d Path optimization measured as a heading was impaired in L7-PP2B mice in dark conditions (repeated measure ANOVA, genotype, light: $F_{1,18}$= 3.64, p=0.073; dark: $F_{1,18}$=8.21, p= 0.010). e Mouse initial orientation, evaluated by the distance between mouse position and platform location after a 100 cm initial trajectory, was altered in L7-PP2B mice in dark conditions (repeated measure ANOVA, genotype, light : $F_{1,18}$= 2.18, p=0.157; dark: $F_{1,18}$=5.61, p= 0.029). f Swim path analyses during both light and dark conditions. The direct trajectory was significantly impaired in L7-PP2B mice during dark conditions (repeated measure ANOVA, genotype, light : $F_{1,18}$= 2.6, p=0.13; dark: $F_{1,18}$=6.7, p= 0.019). Error bars represent S.E.M.

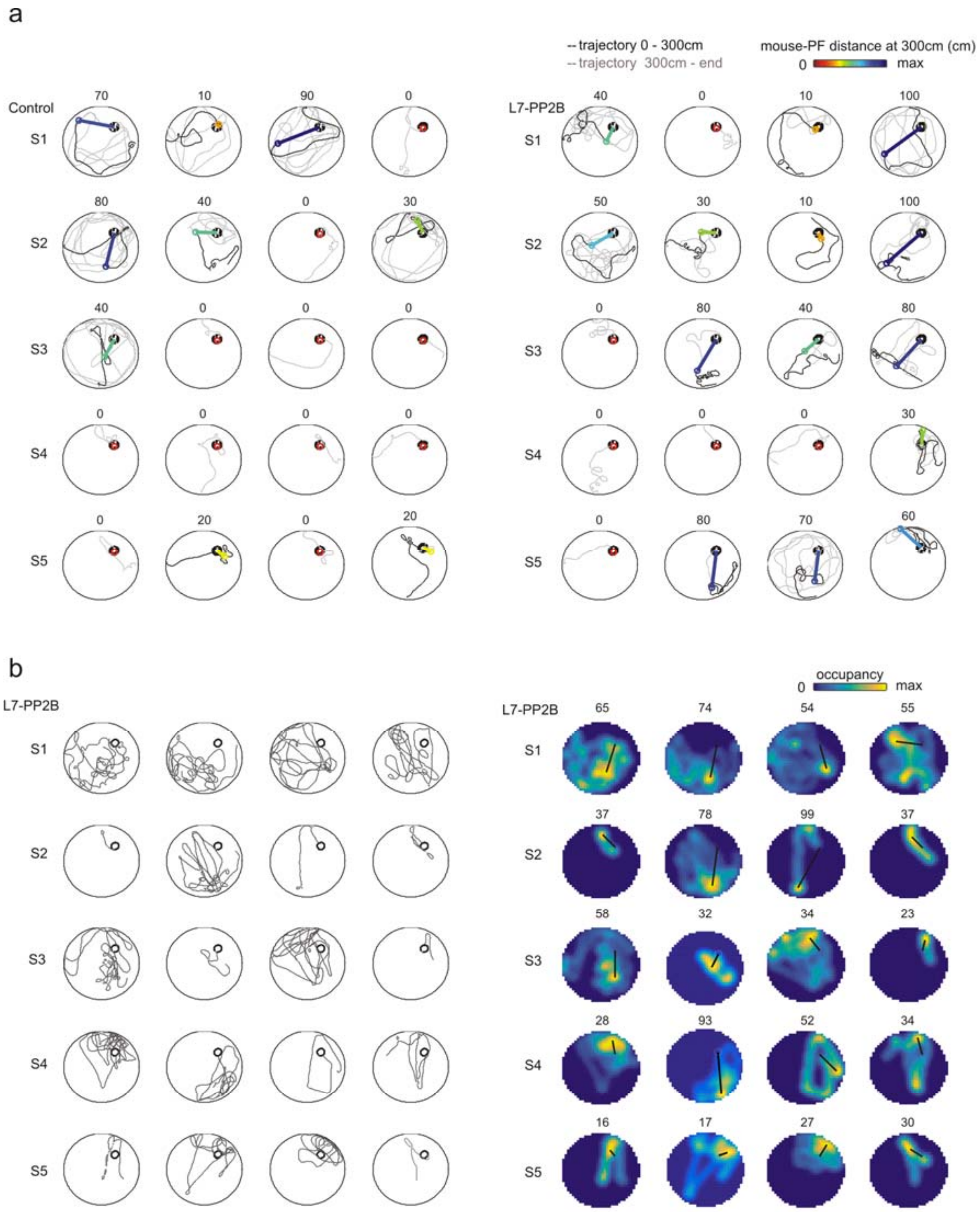

Supplementary Figure 13. Method used to analyze mouse orientation abilities in the Morris Watermaze. a Relevance of mouse initial orientation is measured by the distance between the platform (PF) and mouse location after a 300 cm initial trajectory (presented in Fig. 6d). Example trajectories of a control (left) and a L7-PP2B (right) mouse illustrate the evolution of this measure over the course of training (from session S1 to S5), 4 trials per session). The distance between platform and mouse location after 300cm is highlighted with a bold line whose length is indicated above the trajectories and color-coded for visualization. The initial trajectory (0-300 cm) is indicated in dark grey and the final (300 cm-end) in light grey. b Relevance of mouse search behavior is evaluated by the distance between the peak of single-trial exploration maps and the platform (presented in Fig. 5e). Examples of trajectories left) and associated single-trial maps (right) for a L7-PP2B mouse are presented over the course of training. The distance between the platform and the peak of exploration is highlighted with a black line and its value in cm is indicated above the maps.
