# Supplementary material for: Impaired cerebellar Purkinje cell potentiation generates unstable spatial map orientation and inaccurate navigation
Source: Nat Commun. 2019 May 21;10:2251. doi: 10.1038/s41467-019-09958-5 (PMC6529420; doi:10.1038/s41467-019-09958-5)
Supplement: Supplementary file 3 — Reporting Summary [file 41467_2019_9958_MOESM3_ESM.pdf]

nature research

Corresponding author(s): Laure RONDI-REIG

Last updated by author(s): Mar 26, 2019

# Reporting Summary

Nature Research wishes to improve the reproducibility of the work that we publish. This form provides structure for consistency and transparency in reporting. For further information on Nature Research policies, see Authors & Referees and the Editorial Policy Checklist.

## Statistics

For all statistical analyses, confirm that the following items are present in the figure legend, table legend, main text, or Methods section.

| n/a | Confirmed | |
|---|---|---|
| ☐ | ☒ | The exact sample size ($n$) for each experimental group/condition, given as a discrete number and unit of measurement |
| ☐ | ☒ | A statement on whether measurements were taken from distinct samples or whether the same sample was measured repeatedly |
| ☐ | ☒ | The statistical test(s) used AND whether they are one- or two-sided<br>*Only common tests should be described solely by name; describe more complex techniques in the Methods section.* |
| ☐ | ☒ | A description of all covariates tested |
| ☐ | ☒ | A description of any assumptions or corrections, such as tests of normality and adjustment for multiple comparisons |
| ☐ | ☒ | A full description of the statistical parameters including central tendency (e.g. means) or other basic estimates (e.g. regression coefficient) AND variation (e.g. standard deviation) or associated estimates of uncertainty (e.g. confidence intervals) |
| ☐ | ☒ | For null hypothesis testing, the test statistic (e.g. $F$, $t$, $r$) with confidence intervals, effect sizes, degrees of freedom and $P$ value noted<br>*Give P values as exact values whenever suitable.* |
| ☒ | ☐ | For Bayesian analysis, information on the choice of priors and Markov chain Monte Carlo settings |
| ☒ | ☐ | For hierarchical and complex designs, identification of the appropriate level for tests and full reporting of outcomes |
| ☒ | ☐ | Estimates of effect sizes (e.g. Cohen's $d$, Pearson's $r$), indicating how they were calculated |

*Our web collection on statistics for biologists contains articles on many of the points above.*

## Software and code

Policy information about availability of computer code

| | |
|---|---|
| Data collection | Electrophysiological signals and animal positions were acquired using Datawave SciWorks software (DataWave Technologies, Loveland, CO, USA). The signal go through a manufactured headstage and theter connected to Neuralynx amplifiers (Neuralynx, Bozeman, MT, USA). The signal is stored with DataWave Sciworks acquisition software. Behavioral data acquisition was performed using the SMART® video recording system and tracking software. |
| Data analysis | Spike sorting were made using Offline Sorter software (Plexon).<br>Data analysis was performed using MATLAB programs developed in our laboratory. |

For manuscripts utilizing custom algorithms or software that are central to the research but not yet described in published literature, software must be made available to editors/reviewers. We strongly encourage code deposition in a community repository (e.g. GitHub). See the Nature Research guidelines for submitting code & software for further information.

## Data

Policy information about availability of data

All manuscripts must include a data availability statement. This statement should provide the following information, where applicable:

- Accession codes, unique identifiers, or web links for publicly available datasets
- A list of figures that have associated raw data
- A description of any restrictions on data availability

The data that support the findings of this study are available from the corresponding author upon reasonable request.

# Field-specific reporting

Please select the one below that is the best fit for your research. If you are not sure, read the appropriate sections before making your selection.

☒ Life sciences ☐ Behavioural & social sciences ☐ Ecological, evolutionary & environmental sciences

For a reference copy of the document with all sections, see nature.com/documents/nr-reporting-summary-flat.pdf

# Life sciences study design

All studies must disclose on these points even when the disclosure is negative.

| | |
|---|---|
| Sample size | We choose sample size based on literatures in the field |
| Data exclusions | No data was excluded from the analyses |
| Replication | N/A |
| Randomization | NA. Analyses consisted in comparing genotypes. |
| Blinding | Experiments were carried out in blind conditions with respect to the genotype. |

# Reporting for specific materials, systems and methods

We require information from authors about some types of materials, experimental systems and methods used in many studies. Here, indicate whether each material, system or method listed is relevant to your study. If you are not sure if a list item applies to your research, read the appropriate section before selecting a response.

## Materials & experimental systems

| n/a | Involved in the study | |
|---|---|---|
| ☒ | ☐ | Antibodies |
| ☒ | ☐ | Eukaryotic cell lines |
| ☒ | ☐ | Palaeontology |
| ☐ | ☒ | Animals and other organisms |
| ☒ | ☐ | Human research participants |
| ☒ | ☐ | Clinical data |

## Methods

| n/a | Involved in the study | |
|---|---|---|
| ☒ | ☐ | ChIP-seq |
| ☒ | ☐ | Flow cytometry |
| ☒ | ☐ | MRI-based neuroimaging |

## Animals and other organisms

Policy information about studies involving animals; ARRIVE guidelines recommended for reporting animal research

| | |
|---|---|
| Laboratory animals | The study involved  C57BL//6 mice, transgenic L7-PP2B mice and their wild-type littermates, all from a C57BL/6 mouse strain. |
| Wild animals | The study did not involved wild animals |
| Field-collected samples | The study did not involved field-collected samples |
| Ethics oversight | All data were obtained in compliance with the European and French ethical animal committee (European directive 86-609, French ethical committee n° 5, project 00896.01). |

Note that full information on the approval of the study protocol must also be provided in the manuscript.
